# The COVID-19 Pandemic in Japan

**DOI:** 10.1007/s00595-020-02033-3

**Published:** 2020-05-27

**Authors:** Manabu Watanabe

**Affiliations:** Department of Surgery, Toho University Ohashi Medical Center Manabu Watanabe, 2-22-36 Ohashi, Meguro-ku, Tokyo, 153-8515 Japan

**Keywords:** COVID-19, SARS-CoV-2, Pandemic, Infection control

## Abstract

Since its initial outbreak in China, in December, 2019, COVID-19 has spread rapidly across the globe. At the time of writing, on April 29, 2020, infections had been confirmed in more than 200 countries and regions and 3,018,681 infections and 207,973 deaths had been recorded. In Japan, the first confirmed case of SARS-CoV-2 infection was reported on January 16, 2020, since when, the number of domestic infections and the death toll have reached 13,852 and 389, respectively, representing exponential increases. Moreover, both domestically and internationally, the shortage of medical resources and the spread of infection among medical personnel, caused by nosocomial transmissions have become problematic. The pathology of COVID-19, including the exact infection route, remains largely unknown. Given the unavailability of an effective cure and vaccine, people are required to respond to this adversity without becoming complacent. The global efforts against the COVID-19 pandemic are ongoing endeavors, far from over. New epidemiological data and clinical findings are emerging on a daily basis, making it critical to always refer to the latest information.

## Introduction

The outbreak of pneumonia that originated in Wuhan city in the Chinese province of Hubei in December, 2019 was attributed to a new strain of coronavirus: “severe acute respiratory syndrome coronaviruses 2 (SARS-CoV-2)”, initially called “2019-nCoV” [1, 2]. On February 11, 2020, the World Health Organization (WHO) named the infection caused by SARS-CoV-2 “coronavirus disease 2019 (COVID-19)”. This infection spread quickly from Wuhan to various regions in China, and then to other parts of the world including Japan, Europe, and the United States. On March 11, the WHO officially designated this event a pandemic. At the time of writing on April 29, 2020, a PubMed search of the term “COVID-19” found 7787 articles concerning COVID-19. Given that the first article was published in January this year, the number of submitted papers has been increasing exponentially. The COVID-19 pandemic is an ongoing event across the globe, with the number of confirmed cases increasing rapidly in Japan owing to widespread infection. Amid these circumstances, new reports update the epidemiological information as well as basic/clinical findings on a daily basis. In this manuscript, we report on the documented clinical/surgical findings of COVID-19 as well as the infection status in Japan and other countries.

### New strain of coronavirus: SARS-CoV-2

Based on their genetic characteristics, coronaviruses are classified into four genera: α, β, γ, and δ [3].

Of these coronaviruses, the α-coronaviruses 229E and NL63 and the β-coronaviruses OC43 and HKU1 are known to cause cold symptoms in humans. These four viral strains account for 10%–15% (35% in a peak season) of all cases of the common cold [4]. In the outbreak of severe acute respiratory syndrome (SARS) that began in Guangdong Province, China, humans were infected with a bat-borne coronavirus. Between November, 2002 and July, 2003, the infection spread to more than 30 countries and regions. According to the WHO’s statistics in December 2003, the number of patients with confirmed or suspect SARS reached 8,069, 775 of whom died of severe pneumonia, representing a mortality rate of 9.6%. In 2012, Middle East respiratory syndrome (MERS) was reported in the Arabian Peninsula. This epidemic was caused by camel-to-human infection. As of November 30, 2019, the WHO had confirmed 2494 MERS cases in 27 countries, with 858 deaths, representing a mortality rate of 34.4%.

The SARS-CoV-2 that is causing the current ongoing COVID-19 pandemic belongs to the genus β-coronavirus, the same group as that to which the SARS and MERS pathogens belong [2, 4]. The SARS-CoV-2 strain spreads primarily through respiratory transmission, and its virulence is considered to be lower than that of MERS or SARS. According to the Chinese Center for Disease Control and Prevention, the basic reproduction number (R_0_: the expected number of secondary infections from a single infected individual) of SARS-CoV-2 is 2.2, as estimated from more than 70,000 COVID-19 patients [5]. Although the number varies greatly from report to report (1.4–3.9), it is generally equivalent to or only slightly higher than that of seasonal influenza, showing that the virus is highly contagious among humans [6].

### Clinical pathology of COVID-19

The most frequent symptoms of COVID-19 are fever (94%), cough (79%), muscle pain (15%), and general malaise (23%), and less frequently, diarrhea (5%) [7]. The median period of viral excretion is 20 days [7], which is relatively long, and the median incubation period prior to onset is reported to be 4 days (2–7 days) [8]. Large-scale investigations of Chinese cases have found that while clinical conditions varied widely from asymptomatic to severe pneumonia, 81% were classified as mild (either non-pneumonia or mild pneumonia). Nevertheless, 14% advanced in severity, with 5% requiring intensive care for shock, respiratory failure, or multiple organ failure [9]. The mortality rate of COVID-19 in China was reported as 2.3% (1023/44,672 cases). The risk factors for exacerbation included advanced age and the presence of pre-existing conditions such cardiovascular diseases, diabetes mellitus, chronic respiratory diseases, and malignant tumors. Consequently, the mortality rates were higher for patients with pre-existing conditions, being 10.5% of those with cardiovascular diseases, 7.3% of those with diabetes mellitus, 6.3% of those with chronic respiratory diseases, and 5.6% of those with malignant tumors. Moreover, 81% of deaths were of people aged 60 years or older. People in their 40 s or younger were less likely to suffer exacerbation of their symptoms than those in their 50 s or older. Thus, mortality rates increased with age, being 8.0% for people aged 70–79 years old and 14.8% for people aged 80 years old and older [9–11]. In many severe cases of COVID-19, symptoms often worsened approximately 7 days after onset, with the development of acute respiratory distress syndrome (ARDS) within a few days. The mortality rate for severely affected patients after the development of ARDS was 50% [7, 12] and as high as 61.5% for those with serious SARS-CoV-2 pneumonia requiring intensive care unit (ICU) management. The median period from ICU admission to death was 7 days, indicating an extremely poor prognosis [13]. For this condition, strict ARDS-standard respiratory management is implemented, with extracorporeal membrane oxygenation (ECMO) in patients with even more serious respiratory failure. However, the decision to initiate ECMO requires the judgment of highly experienced acute care specialists. In Japan, organizations such as the Japanese Society of Intensive Care Medicine and the Japanese Association for Acute Medicine jointly launched a taskforce called “Japan ECMO net for COVID-19,” thereby extending support to ECMO-based treatments for cases of COVID-19-related serious respiratory failure. ECMO application needs to be decided on carefully and comprehensively. The use of ECMO is contraindicated for patients with irreversible underlying disease or terminal-stage cancer as well as for patients aged 75 or older because of the associated poor prognosis. As of April 20, 2020, 90 patients had received ECMO treatment for COVID-19-related serious respiratory failure in Japan. Of the 52 patients who completed ECMO treatment, 35 recovered (67%) and 17 died (33%) [14]. While the efficaciousness of ECMO is promising, patients with severe pulmonary fibrosis may need to be withdrawn. COVID-19-related serious respiratory failure is characterized by rapid aggravation and slow recovery. Nevertheless, given that ECMO compliance is often favorable, it is imperative that ECMO treatment be initiated without delay.

There is still no effective cure for COVID-19 or a practical vaccine. The basic therapeutic modality is anti-infection control and supportive therapy for pneumonia and other conditions [15]. Possible candidate drugs include anti-HIV lopinavir/ritonavir, anti-influenza favipiravir (tradename: Avigan), anti-Ebola remdesivir, and inhaled corticosteroid ciclesonide. Provided that further studies substantiate their efficacy, they may be used as curatives. Notably, it was reported that 36 of 53 COVID-19 patients who received treatment with remdesivir showed signs of clinical improvement, raising expectations that it may be used as an effective curative in the future [16].

### COVID-19-related findings in the field of surgery

With the global spread of COVID-19, it is expected that more SARS-CoV-2-postive patients in Japan will need to undergo surgical procedures. To ensure that the patients are safe, medical personnel and medical instruments are not exposed, and nosocomial infections are prevented, relevant organizations must urgently formulate guidelines for handling SARS-CoV-2-positive patients during periprocedural periods. Against this backdrop, surgery-related academic societies, including the Japan Surgical Society, compiled precautions for surgeons and published a proposal on April 1, 2020 [17]. Please refer to other articles on COVID-19-related clinical issues in surgery for various organs. In this manuscript, we elaborate on two issues relating to the entire surgical field: “postponement of elective surgery” and “surgical smoke and infection control.”

#### Postponement of elective surgery

Many surgical academic societies, including the Japan Surgical Society and the American College of Surgeons, recommend that given the current global spread of COVID-19, surgical treatment be limited to patients requiring emergency surgery for life-threatening conditions, and that elective surgery for patients with non-fatal or non-urgent disease be postponed [17, 18]. Each medical institution should give multifaceted consideration to whether elective surgery should be implemented or postponed, not only to prevent risk but also from the perspectives of medical necessity and effective and efficient distribution of medical resources. Postponement of elective surgery should minimize not only the infection risk for both patients and medical personnel, but also the use of necessary medical resources such as beds, artificial ventilators, and personal protective equipment (PPE) [19, 20]. There have been several reports on the risks of surgery for COVID-19 patients. In a study on 34 asymptomatic COVID-19 patients, all developed COVID-19-induced pneumonia immediately following surgery, 15 (44.1%) of whom required ICU treatment [21]. This percentage (44.1%) was far higher than the 26.1% of non-surgically treated COVID-19 patients. Furthermore, seven patients admitted to the ICU (20.6%) died of COVID-19-related complications [22], and another report states that three of four patients who developed COVID-19 perioperatively to elective surgery died [23]. These findings support that elective surgery should be postponed as much as possible in areas of a COVID-19 outbreak [24]. When surgery or emergency procedures that cannot be deferred need to be performed for a COVID-19 patient, a careful plan must be implemented [25]. It is also important that all patients scheduled for surgery are tested preoperatively for SARS-CoV-2 infection [26].

With Japan seeing an increasing number of COVID-19 cases, surgical indications should be fully assessed to ensure safe treatment, and if surgery is deemed necessary, all conceivable measures must be taken to alleviate risks of infection during the management and periprocedural control of the operating theater.

#### Surgical smoke and infection control

Surgical smoke refers to fumes emitted from energy devices and consists of diverse airborne particles in the atmosphere or the body cavity. It contains various substances, including viral and carcinogenic compounds [27]. Previous studies have identified that laparoscopy may cause aerosolization of blood-derived viruses [28, 29]. Although the infection risk of surgical smoke generated during surgery on a COVID-19 patient has not been fully assessed, countermeasures should be prepared on the premise that surgical smoke contains SARS-CoV-2. Risks of viral aerosol infection have been shown to be explicitly higher following laparoscopic surgery than conventional laparotomy. Accordingly, the use of high-precision filters and exhaust gas apparatus must be implemented in laparoscopic surgery. Additionally, intraabdominal pressure and CO_2_ insufflation should be kept as low as possible during surgery [30], and electric surgical units should also be at the lowest settings able to achieve the desired effects [31, 32].

Overall, 8%–15% of patients infected with SARS-CoV-2 have experienced viremia [33–35], with viral presence confirmed in urine and fecal samples [36]. Accordingly, care is required in implementing general regimens, including endoscopic examination, treatment, and handling of body fluids. In responding to the pandemic, it must be kept in mind that SARS-CoV-2 remains infectious for several days on the surfaces of various objects [37]. Because contamination may not be contained within operating theaters, hospitals need to take preventive measures. Surgeons must also ensure that anti-infection strategies are in place, while always being aware that aerosol, droplet, and contact infections from patients infected with SARS-CoV-2 are serious risk factors for their safety.

### Infection status in Japan and abroad

**Fig. 1:**
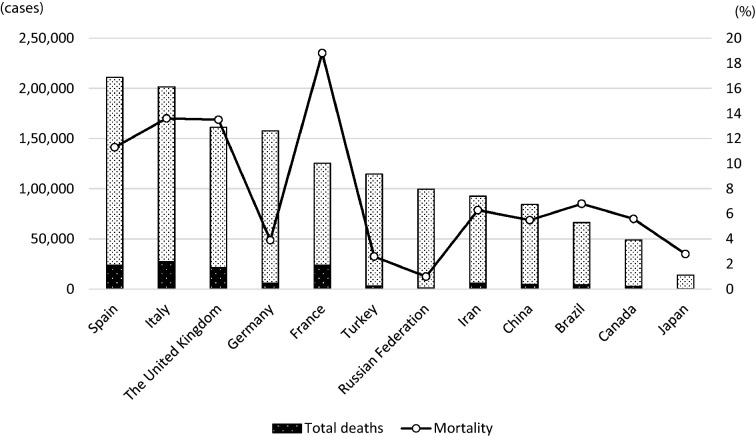
Confirmed number of COVID-19 cases and mortality worldwide. Data as of April 29, 2020

COVID-19 has spread rapidly across the world since its original outbreak in China in December, 2019. At the time of writing, on April 29, 2020, infections had been confirmed in more than 200 countries and regions, with 3,018,681 infections and 207,973 deaths reported (mortality rate: 6.9%) [38]. Figure 1 shows the countries with large numbers of infections. At the initial stage of the pandemic, the overwhelming majority of cases were in China; however, the number of new cases in Mainland China has been decreasing. Currently, the number of infections recorded has exceeded 100,000 in the following seven countries: The United States, Spain, Italy, The United Kingdom, Germany, France, and Turkey. While the majority are European countries, the US has recorded the largest number of infections (983,457) and deaths (50,492) (mortality rate; 5.1%), showing an exponential increase in the 2 months from March 2, 2020, when 69 infections and 1 death were recorded. Overall, the US accounts for 32.6% of the global infections and 24.3% of the global death toll. While France reports the highest mortality rate of 18.8% (23,627/125,464 infections), the New York metropolitan area saw 5700 newly hospitalized COVID-19 patients during the month of March 2020. Of the 2634 patients who completed treatment, 553 (21%) reportedly died [39].

**Fig. 2:**
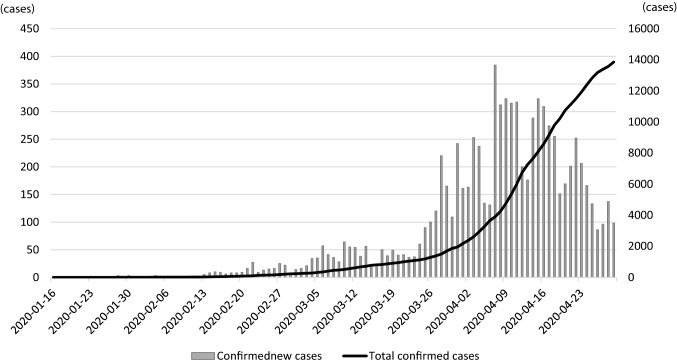
Confirmed number of COVID-19 cases in Japan (January 16, 2020 - April 29, 2020)

**Fig. 3:**
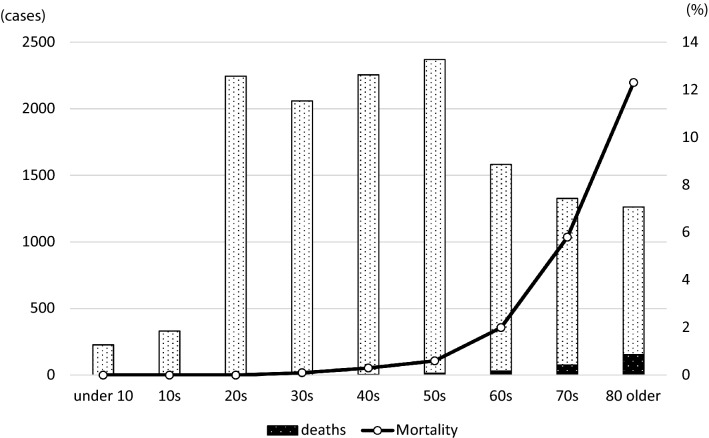
Confirmed number of COVID-19 cases and mortality, by age group, in Japan. Data as of April 29, 2020

In Japan, the first confirmed case of SARS-CoV-2 infection was recorded on January 16, 2020, when a Chinese national who had visited Wuhan tested positive [40]. On February 3, 2020, many of the 3711 passengers and crew members of a cruise liner named “Diamond Princess” were found to be infected when the ship docked in the Port of Yokohama. Subsequently, the number of infected individuals amounted to 712 (19.2%), 13 of whom died. The basic reproduction number (R_0_) of the virus among the passengers of the Diamond Princess was initially estimated to be 14.8, indicating a highly contagious environment. If no quarantine had been enforced, it is predicted that 79% (2920 people) would have become infected. However, on account of the timely disembarking and quarantine measures, the R_0_ decreased to 1.78. Compared with a situation where there was no intervention, these efforts were estimated to have prevented more than 2000 additional infections [41]. As of March 2, 2020, the number of domestic infections was 239 and the death toll was 6, excluding those detected aboard the Diamond Princess. The number of cases started growing rapidly, mainly in urban areas. At the time of writing, the number of domestic infections and the death toll had reached 13,852 and 389, respectively, showing exponential increases (Fig. 2). However, the mortality rate has remained relatively low at 2.8% [42]. By age group, those aged 80 or older have the highest mortality rate of 12.3% (155/1263 infections); significantly higher than that of those in their 70 s (5.8%; 77/1327 infections; Fig. 3).

Both domestically and internationally, the shortage of medical resources and the spread of infection among medical personnel from nosocomial transmissions have become problematic [43]. One Chinese report states that 57 of 138 COVID-19 patients (41.3%) may have caught the disease in a hospital and that 40 of these patients (29%) were medical personnel, of whom 31 (77.5%), 7 (17.5%), and 2 (5%) were working in a general ward, emergency department, and ICU, respectively [22]. When the mortality of COVID-19 and the cumulative number of cases per population are plotted, a significant positive correlation is observed, revealing a correlation between the mortality and medical expenses [44]. This indicates that when an outbreak occurs in highly populated areas such as big cities, an increase in the cumulative number of patients per population will result in further deprivation of medical resources. As medical resources are depleted, more medical personnel will be infected. This scenario must be avoided at all costs if we are to prevent a total collapse of the medical care system. COVID-19 poses a great threat to all medical practitioners. As such, thorough preventive measures must be taken against standard, contact, and droplet infections to ensure safe diagnostic care is given to all patients. Strict measures against nosocomial infections should also be implemented and early detection of an infection and the appropriate response are crucial to prevent further transmission.

## Conclusion

Much of the pathology of COVID-19 remains unknown, including the exact infection route. Given the unavailability of effective cures and vaccines, people are required to respond to this adversity without becoming complacent. The National Center for Global Health and Medicine has been engaged in observational research by enrolling COVID-9 patients and this new registry may be used for analyzing serious domestic cases, their characteristics, course, and efficacy of treatment. We hope that the new findings will serve as basic data for the development of treatments and new drugs. The global efforts in combatting the COVID-19 pandemic are ongoing and far from over. New epidemiological data and clinical findings are emerging each day, making it critical to always refer to the latest information. We hope that the COVID-19 pandemic will have shown signs of containment by the time this manuscript is published.
